# Impact of molecular profiling on overall survival of patients with advanced ovarian cancer

**DOI:** 10.18632/oncotarget.7835

**Published:** 2016-03-01

**Authors:** Thomas J. Herzog, David Spetzler, Nick Xiao, Ken Burnett, Todd Maney, Andreas Voss, Sandeep Reddy, Robert Burger, Thomas Krivak, Matthew Powell, Michael Friedlander, William McGuire

**Affiliations:** ^1^ University of Cincinnati Cancer Institute, Cincinnati, OH, USA; ^2^ Caris Life Sciences, Phoenix, AZ, USA; ^3^ University of Pennsylvania, Philadelphia, PA, USA; ^4^ Western Pennsylvania Gynecological Oncology, Mars, PA, USA; ^5^ Washington University School of Medicine, St. Louis, MO, USA; ^6^ Prince of Wales Hospital, Sydney, Australia; ^7^ VCU Massey Cancer Center, Richmond, VA, USA

**Keywords:** ovarian, cancer, molecular, profiling, survival

## Abstract

**Objective:**

Patients with recurrent epithelial ovarian cancer (EOC) have limited treatment options. Studies have reported that biomarker profiling may help predict patient response to available treatments. This study sought to determine the value of biomarker profiling in recurrent EOC.

**Results:**

Patients in the Matched cohort had a median OS of 36 months compared to 27 months for patients in the Unmatched cohort (HR 0.62, 95% CI 0.41-0.96; p < 0.03). Individual biomarkers were analyzed, with TUBB3, and PGP prognostic for survival. Biomarker analysis also identified a molecular subtype (positive for at least two of the following markers: ERCC1, RRM1, TUBB3, PGP) with particularly poor overall survival.

**Methods:**

224 patients from a commercial registry (NCT02678754) with stage IIIC/IV EOC at diagnosis, or restaged to IIIC/IV EOC at the time of molecular profiling, were retrospectively divided into two cohorts based on whether or not the drugs they received matched their profile recommendations. The Matched cohort received no drugs predicted to be lack-of-benefit while the Unmatched cohort received at least one drug predicted to be lack-of-benefit. Profile biomarker/drug associations were based on multiple test platforms including immunohistochemistry, fluorescent *in situ* hybridization and DNA sequencing.

**Conclusions:**

This report demonstrates the ability of multi-platform molecular profiling to identify EOC patients at risk of inferior survival. It also suggests a potential beneficial role of avoidance of lack-of-benefit therapies which, when administered, resulted in decreased survival relative to patients who received only therapies predicted to be of benefit.

## INTRODUCTION

Almost a quarter of a million women worldwide are diagnosed each year with epithelial ovarian cancer (EOC; including primary peritoneal and fallopian tube carcinomas) and it is the leading cause of gynecologic cancer-related death in developed countries. The 5-year survival of EOC patients is only 44% due to the fact that 75% of women present with advanced disease [1]. Approximately 80% of advanced stage patients who have residual disease after surgery and receive front-line platinum-based combination chemotherapy respond and experience a median progression free survival (PFS) of 18 months [2]. Patients who have recurrence of disease within 6 months of completion of their initial treatments have been traditionally classified as platinum-resistant. Fewer than 15% of these patients typically respond to the next line of treatment and have a median survival of less than a year [3]. Most cases that are initially platinum-sensitive eventually develop platinum resistance.

There are a variety of EOC histologies with distinct molecular profiles and clinical courses [1, 4]. For example, high-grade serous ovarian cancer (HGSC) accounts for the majority of cases and a disproportionate number of deaths. Other subtypes, such as clear cell or mucinous ovarian carcinomas, are less common and have poor responses to standard therapies used to treat HGSC. Additionally, large-scale gene expression analyses have identified molecular subtypes within HGSC with variable survival rates [5] and degrees of platinum resistance [6].

These variations in treatment response underscore the need for molecular characterization of EOC in order to identify treatment options that are most likely to benefit individual patients. Some published evidence supports the use of DNA repair proteins (ERCC1, BRCA1/2) as markers for platinum response; however, there are conflicting data [5-7]. RRM1, TUBB3/PGP and TOP2A have been shown to be predictive of response to gemcitabine, taxanes and anthracyclines, respectively, and represent drugs commonly used to treat patients with EOC [8-12]. Beyond TP53 and BRCA, few mutations commonly exist in EOC [13]; however, broad sequencing has the potential to identify rare mutations associated with potentially effective therapies not typically considered in this disease.

Studies have shown that multiplatform molecular profiling (profiling considering both protein and DNA abnormalities) has clinical utility in a variety of cancer types. A recent study in patients with refractory breast cancer showed that tumor profiling resulted in a revision of the original treatment decision for every patient, and tumor profiling-based therapy resulted in clinical benefit for 52% of the 25 heavily pretreated patients [14]. A multi-lineage pilot study showed that comprehensive molecular profiling identified molecular targets in patients with refractory metastatic cancer of multiple histogenetic types [15]. In this study, 18 of 66 patients treated with a molecularly guided therapy had a longer PFS as compared to their prior PFS interval with treatment chosen without molecular guidance. Subsequent studies have demonstrated the benefit of multiplatform profiling in other clinical settings in other tumor types as well [16, 17].

Thus, the evidence suggests that multi-platform tumor profiling has the potential to assist in clinical decision-making and increase the likelihood of response to chemotherapy in patients with recurrent EOC. To evaluate the effectiveness of one such profile, we evaluated clinical data from the Caris observational Registry, whereby patient molecular profiling data were collected and coupled with clinical outcomes recorded in a central database. The impact of profiling on drug usage, median survival and overall survival (OS) was assessed. The contribution of individual biomarkers was also measured.

## RESULTS

### Patient characteristics

**Figure 1:**
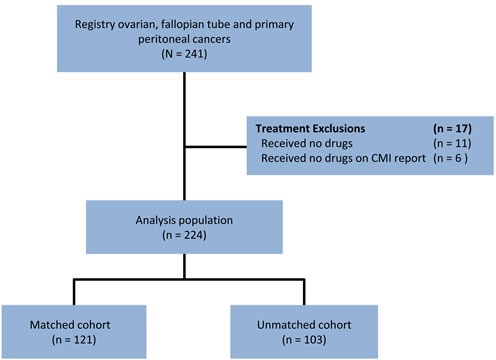
Retrospective analytic schema

There were 241 EOC patients with advanced stage cancers who underwent treatment and had at least 9 months of follow-up data, diagnostic staging of at least IIIC or having metastasis or treatments prior to profiling. Of 241 eligible patients, 17 were excluded because they received no drugs after the time of tissue collection or the drugs they received were not classified by the molecular profile (received no drugs of predicted benefit or lack-of-benefit; Figure 1).

The analysis population of 224 patients was divided into two cohorts based on the matching of treatments to profile recommendations. Dividing the group into only two cohorts provided a straightforward approach to answer the key question: Do patients whose treatments consistently follow profile results do better than patients whose treatments are inconsistent with profile results? Grouping into only two cohorts also allowed for statistical power to make significant claims regarding observed differences.

The Matched cohort (n=121) includes patients who received at least one treatment associated with potential benefit and no treatments associated with lack-of-benefit at any time following the date of sample collection for the specimen submitted for profiling. The Unmatched cohort (n=103) includes patients who received at least one treatment associated with potential lack-of-benefit at any time following the sample collection date.

**Figure 2:**
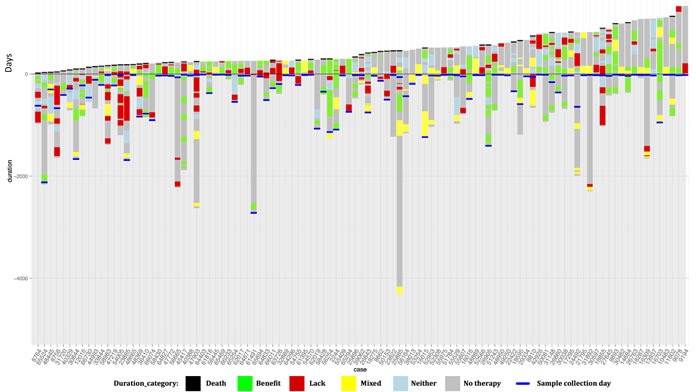
Plots showing duration of monitoring, duration of treatments received before and after profiling, and post-profiling survival for each patient in the study Each column along the x-axis represents one patient. The y-axis is time (days). The zero point of the y-axis is the time of profiling. Patients are sorted left to right based upon survival time post-profiling. Grey bars represent the total time monitored, from diagnosis to either death or last follow-up. Black bars at the top of a column represent death. The colored bars represent drug treatments and are coded relative to their match status with the patient's molecular profile. Green bars represent time on a therapy associated with benefit. Red bars represent time on a therapy associated with lack-of-benefit. Yellow bars represent time on a combination regimen associated with both benefit and lack-of-benefit. Blue bars represent time on a therapy associated with neither benefit nor lack-of-benefit. Panel A shows patients in the Matched cohort, and Panel B shows patients in the Unmatched cohort.

Only drugs administered after the date of collection of the profiled specimen were used to sort the patients into the two cohorts. This method sorted 6 patients into the Matched group who received lack-of-benefit therapies prior to the profiled sample collection date (patients with red and/or yellow treatment boxes in Figure 2A). Sorting of these patients into the Matched group did not significantly impact the study results. Physician consultations were not a part of this study, so no information could be provided regarding why patients were or were not treated according to profile recommendations.

Patient characteristics (age, race, biopsy site, stage at diagnosis and histology) across Matched and Unmatched cohorts are shown in Table 1. The Matched cohort differed substantially in two categories: stage IC at diagnosis (8.3% vs. 2.9%) and not-otherwise-specified (NOS) histology (9.9% vs. 1.0%). The stage IC Matched cohort median survival was lower than the survival for the IC Unmatched cohort, indicating that the Matched IC group was not contributing bias towards longer survival. Resistant histologies (clear cell, small cell and mucinous) were more common in the Unmatched cohort, however, the survival distribution of these cases did not significantly bias OS.

**Table 1:** Demographics of Matched and Unmatched cohorts

Characteristic	Matched n = 121 (%)	Unmatched n = 103 (%)
*Age*		
<40	2 (1.7)	2 (1.9)
40-49	19 (15.7)	17 (16.5)
50-59	33 (27.3)	31 (30.1)
60-69	37 (30.6)	31 (30.1)
70-100	30 (24.8)	22 (21.4)
*Race*		
White	110 (90.9)	91 (88.3)
Black	3 (2.5)	8 (7.8)
Asian	6 (5.0)	3 (2.9)
Other/Unknown	2 (1.7)	1 (1.0)
*Primary Site*		
Ovary	103 (85.1)	86 (83.5)
Fallopian tube	9 (7.4)	5 (4.9)
Peritoneum	9 (7.4)	12 (11.7)
*Stage at Diagnosis*		
I-IA	3 (2.5)	3 (2.9)
IC	10 (8.3)	3 (2.9)
IIA	2 (1.6)	2 (1.9)
IIB	3 (2.5)	3 (2.9)
IIC	1 (0.8)	1 (1.0)
III-IIIA	2 (1.6)	5 (4.9)
IIIB	3 (2.5)	3 (2.9)
IIIC	78 (64.5)	72 (69.9)
IV	13 (10.7)	9 (8.7)
Unknown	6 (5.0)	2 (1.9)
*Histology*		
Serous	91 (75.2)	85 (82.5)
Endometrioid	9 (7.4)	3 (2.9)
Mixed Cell	3 (2.5)	6 (5.8)
Clear Cell	3 (2.5)	5 (4.9)
Mucinous	1 (0.8)	2 (1.9)
Transitional Cell	2 (1.7)	0 (0.0)
Small Cell	0 (0.0)	1 (1.0)
Carcinoma, NOS/Adenocarcinoma	12 (9.9)	1 (1.0)

### Treatment analysis

Waterfall plots were constructed for both study cohorts in order to visualize individual patient monitoring times, treatment durations, and post-profiling survival (Figure 2). Patients are stratified from left to right by post-profiling survival time. Green bars indicate drugs received predicted to be of benefit while red bars indicate drugs predicted to be of lack-of-benefit. Yellow bars indicate times where the patient received drugs predicted to be of both benefit and lack-of-benefit at the same time. In the Matched cohort, 31% of patients are deceased vs. 46% of the Unmatched cohort patients. The median follow-up time for the Matched cohort was 475 days vs. 372 days for the Unmatched cohort.

**Figure 3:**
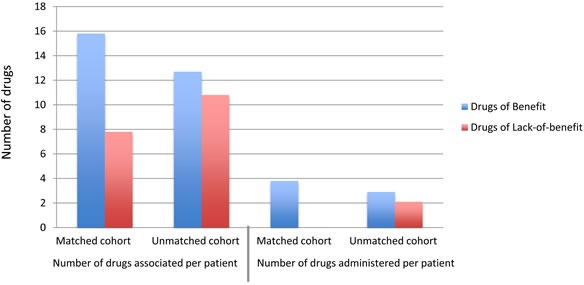
The average number of drugs that were predicted to be of benefit (blue) or lack-of-benefit (red) for each cohort compared to the average number of drugs that the patients actually received (calculated from diagnosis)

The most frequently administered chemotherapy agents with their associated biomarker frequency distribution are presented in Table 1. Patients in the Matched cohort received a median of 3.88 lines of therapy vs. 5.02 lines for patients in the Unmatched cohort (calculated as therapies administered after diagnosis; Figure 3). 83% of patients in the Unmatched cohort received at least one drug predicted to be of benefit, while 56% received two or more benefit drugs. This highlights the fact that the most significant distinguishing factor between the two cohorts is the difference in administration of lack-of-benefit drugs, not benefit drugs. Several administered drugs did not have a recommendation for or against and appear in the “neither” category. This “neither” category makes up 27% of drugs administered in the Matched cohort vs. 30% in the Unmatched cohort. Two common agents in the “neither” category were bevacizumab (given to 34% patients) and cyclophosphamide (given to 5% of patients). These drugs are not included in Table 2 as the molecular profiling panel studied in this report does not associate a biomarker with either drug.

**Table 2:** Number and frequency of results for notable biomarkers

Drug or drug class	Biomarker	Patients tested	Number of patients predicted to benefit (n)*	Number of patients predicted to not benefit (n)*	Number predicted to benefit who received the drug (%)	Number predicted to not benefit who received drug (%)
Platinum	ERCC1	176	143	33	133 (93.0%)	31 (93.9%)
Taxane	TUBB3	163	92	74	80 (87.0%)	63 (85.1%)
	PGP	200	185	17	159 (79.5%)	14 (82.4%)
Gemcitabine	RRM1	207	155	55	61 (39.4%)	11 (20.0%)
Liposomal doxorubicin	TOP2A	182	124	63	42 (33.9%)	14 (22.2%)
Topotecan	TOPO1	207	103	106	18 (17.5%)	18 (17.0%)

* Some cases have been profiled multiple times and have both positive and negative results but do not have conflicting therapy associations

Table 2 also reports the frequency of administration of drugs that were predicted to be of benefit or lack-of-benefit for the cohorts. As expected, drugs most commonly used to treat EOC were administered at similar rates whether or not they were predicted to be of benefit to the patient (platinum agents and taxanes): 94% and 85% of patients who were predicted not to benefit from platinum and taxanes, respectively, received the agents anyway. On the other hand, gemcitabine and doxorubicin were less likely to be administered to patients who were predicted to not benefit from these drugs (only 20% and 22% received gemcitabine or doxorubicin with a lack-of-benefit prediction).

Drug/biomarker associations in the profile include both on and off-label drugs for ovarian cancer. Thus, many patients received results for drugs outside of the National Comprehensive Cancer Network guidelines for treatment of EOC (on-compendium vs. off-compendium). 12% of patients in the Matched cohort (14/121) received an off-compendium agent compared to 23% in the Unmatched cohort (24/103). The majority (68%) of off-compendium agents administered were not associated with a profile biomarker and were classified in the “neither” category (26/38). Few physicians used the profile results to prescribe off-compendium drugs predicted to be of benefit (only three patients in each arm).

### Survival analysis

**Figure 4:**
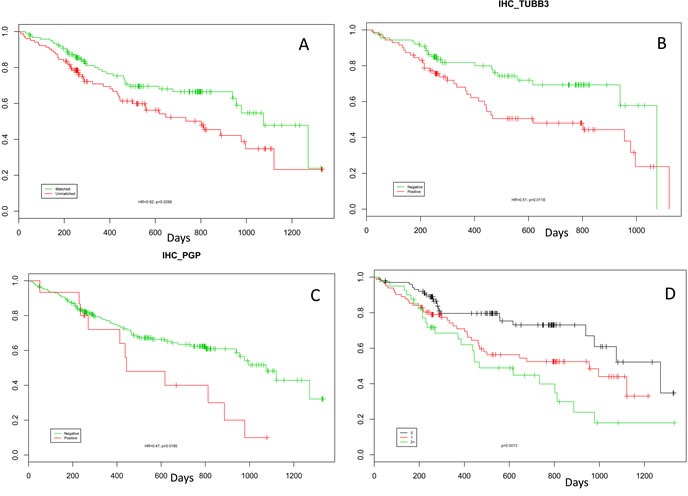
Kaplan-Meier curves **A.** Kaplan-Meier curve showing the increase in overall survival from time of profiling for those patients treated only with therapies predicted to be of benefit by their molecular profile compared to those patients who received at least one therapy predicted be lack-of-benefit (HR 0.62, p=0.0295). Kaplan-Meier curves for patients who were positive for TUBB3 (**B**) and PGP (**C**). **D**: Kaplan-Meier curves showing that patients with over-expression of multiple markers have decreased overall survival. The green line shows patients who were positive for two or more biomarkers from the set ERCC1, PGP, RRM1, and TUBB3. The red line shows patients who were positive for only one biomarker from this set. The black line shows patients who were not positive for any biomarkers in this set.

Patients in the Matched cohort experienced a significantly greater improvement in OS from the time of molecular profiling when compared to patients in the Unmatched cohort. Median OS from the time of tumor profiling for patients included in the Matched cohort was 36 months compared to 27 months for patients in the Unmatched cohort (HR 0.62, 95% CI 0.41-0.96; p < 0.03; Figure 4A). Patients who received more than one drug in the lack-of-benefit category trended towards worse OS than patients who received only a single drug in this category (data not shown). Median OS from the time of diagnosis for patients in the Matched cohort was 80 months compared to 56 months for patients in the Unmatched cohort (HR 0.65, 95% CI 0.43-0.99; p=0.045).

Notable biomarkers with demonstrable differences between the Matched and Unmatched cohorts include ERCC1, TUBB3, and PGP. Levels of the taxane-resistance markers PGP (HR 0.47, 95% CI 0.25-0.89, p=0.019) and TUBB3 (HR 0.51, 95% CI 0.30-0.87, p=0.012) were significantly different between the two cohorts (Figure 4B and 4C). While not significant, patients with low ERCC1 by IHC had improved OS (HR 0.62, 95% CI 0.36-1.06, p=0.08). Each of these markers shows a similar trend whereby patients with increased expression have worse OS relative to patients that do not overexpress the proteins. In addition, OS decreased for patients who had more than one of these discrepant biomarkers. Patients positive for either one or more than one of these markers had significantly lower OS than patients who were not positive for any of the markers (Figure 4D).

## DISCUSSION

This initial report evaluating molecularly profiled patients enrolled in an observational registry demonstrates improved OS in EOC patients treated with agents of potential benefit when compared to patients who received agents associated with lack-of-benefit. Further analysis of the biomarker-drug combinations revealed that patients in the Unmatched cohort were less likely to benefit from platinum and taxanes, therapies commonly given to EOC patients, both in front-line and recurrent settings. This suggests that these markers (ERCC1, TUBB3 and PGP) are prognostic and could be predictive; however, almost every woman in this study was treated with a platinum/taxane combination therapy, thus confounding the predictive utility of the individual markers.

Unsurprisingly, the vast majority of patients received platinum and taxanes regardless of whether the profile report predicted a benefit from the drug. While this would be expected no matter when in the course of the disease the profile report was received, most of these patients were profiled following the application of primary therapy. In contrast to platinums and taxanes, gemcitabine and doxorubicin were more likely to be given only when the profile report recommended benefit, suggesting that, once patients were in the platinum-resistant state, physicians were using the profile to direct salvage therapy.

A minority of patients in each cohort received non-NCCN guideline agents, however, most of these drugs were of the “neither” category, indicating that the profile provided no predictive data for these agents. The Unmatched cohort received more off-compendium agents than the Matched cohort. Of the off-compendium agents that were associated with a profiled biomarker, the majority of agents were administered against the profile recommendation, suggesting that physicians were following the profile results less often as their patients' diseases advanced (i.e., trying unconventional treatments in desperate patients regardless of molecular evidence). There were no clear trends in off-compendium agents administered.

The Unmatched cohort received 1.2 more lines of therapy than the Matched cohort and experienced inferior OS. One explanation for this finding is that it is the result of inherent biologic selection of an intrinsically resistant phenotype, which would be expected to be resistant to multiple additional therapies. This is supported by the observation that the ratio of benefit to lack-of-benefit drugs in the Matched cohort was 2.01 while the ratio of benefit to lack-of-benefit drugs in the Unmatched cohort was 1.17. However, the survival curves from time of diagnosis initially overlap and then diverge after profiling occurs. This type of divergence would suggest that basing therapy on tumor profiling has an effect on selecting optimal therapies and improving OS.

Independent biomarker analysis identified the phenotype of a particularly poor-performing subset of patients. This poor-performing phenotype was defined as having high levels of at least two markers in the set of ERCC1, PGP, RRM1, and TUBB3 (double-positive EOC). Our data identified 24% of patients as having this phenotype (of 86 cases who had all 4 markers tested), with a higher concentration in the Unmatched cohort (Matched 10% and Unmatched 45%). We believe this group represents a previously unrecognized subtype of EOC patients who experience inferior OS when treated with the standard-of-care platinum/taxane combination and should be identified early for alternative treatments such as liposomal doxorubicin, topotecan, cyclophosphamide, bevacizumab or a clinical trial. We do not believe that this phenotype is simply a function of the profiling of previously treated, resistant tumors, as 72% of the double-positive-or-higher arm (Figure 4D) were profiled using treatment-naïve samples (56% of the quadruple-negative arm samples were treatment-naïve).

In any prospective observational study, there can be significant sources of bias that diminish the strength of the conclusions obtained from the data. This study is no different, in that even though the cohorts were well balanced, there was no randomization to control for unknown sources of bias. Performing a randomized study in this population would be challenging as the required numbers for enrollment would be high and the follow-up time for OS would be 5-10 years. Additionally, there could be ethical concerns of randomizing patients to a non-profiled arm, as many EOC patients receive some amount of profiling as part of their routine care. The data do allow for a number of potential sources of bias to be eliminated, specifically age, race, stage, histology, grade, and site of biopsy. Other potential sources of bias include physicians self-selecting patients for the study by choosing to profile some patients versus others and physicians choosing to follow or not follow the biomarker recommendations based on unrecorded patient characteristics. The data also show heavy censoring early in the Kaplan-Meier curves for both patient cohorts, which reflects the nine-month minimum follow-up window and the immature clinical follow-up of this Registry. However, the median follow-up time was over a year, and median survival was reached for both cohorts, suggesting that the maturity of the Registry was sufficient for the analyses performed in this study.

In conclusion, this report suggests a potential predictive role of molecular profiling to avoid use of inactive therapies. Additionally, a prognostic biomarker-derived phenotype was identified that demonstrated particularly inferior OS. The conclusions generated here, while intriguing, will need to be validated in an additional prospective observational study with much larger patient numbers.

## MATERIALS AND METHODS

### Molecular techniques

Molecular profiling was performed using a multiplatform approach (Caris Life Sciences® Molecular Intelligence™ [CMI™]) to stratify agents by degree of potential therapeutic benefit. Tumor biopsy samples were analyzed with a combination of Sanger sequencing, next generation sequencing, pyrosequencing, immunohistochemistry (IHC), gene amplification with fluorescent/chromogenic in-situ hybridization (F/C-ISH), and ribonucleic acid fragment analysis depending on physician request.

IHC analysis was performed on formalin-fixed paraffin-embedded tumor samples using commercially available detection kits, automated staining techniques (Benchmark X, Ventana, AutostainerLink 48, Dako), and commercially available antibodies: ERCC1 (8F1, Abcam), RRM1 (10526-1-AP, Proteintech), PGP (C494, Invitrogen), TUBB3 (PRB-435P, BioLegend; see supplemental materials for full list of antibodies).

FISH was used for evaluation of HER-2/neu [HER-2/CEP17 probe], EGFR [EGFR/CEP7 probe], and cMET [cMET/CEP7 probe] (Abbott Molecular/Vysis). HER-2/neu and cMET status were evaluated by CISH (INFORM HER-2 Dual ISH DNA Probe; commercially available cMET and chromosome 7 DIG probe; Ventana). The same scoring system was applied as for FISH.

Direct sequence analysis was performed on genomic DNA isolated from formalin-fixed paraffin-embedded tumor samples using the Illumina MiSeq platform. Specific regions of 45 genes of the genome were amplified using the Illumina TruSeq Amplicon Cancer Hotspot panel. Mutation analysis by Sanger sequencing included selected regions of BRAF, KRAS, c-KIT, EGFR, and PIK3CA genes and was performed by using M13-linked PCR primers designed to amplify targeted sequences.

### Statistical considerations

The Caris Registry (NCT02678754) was queried for all patients with a diagnosis of ovarian, primary peritoneal and fallopian tube carcinomas enrolled between 2010 and 2014. This IRB-approved prospective observational study includes baseline clinical information at the time of profiling (not necessarily at the time the pathologic material was obtained), profiling results, treatments received and clinical outcomes, including PFS and OS, updated at nine-month intervals after enrollment (as specified in the Registry protocol). Survival was calculated from the date of molecular profiling and from the date of diagnosis to the date of death or last follow-up date. OS from the date of molecular profiling was chosen as the primary endpoint.
